# Predictors for long‐term mortality in COPD patients requiring non‐invasive positive pressure ventilation for the treatment of acute respiratory failure

**DOI:** 10.1111/crj.13251

**Published:** 2020-09-22

**Authors:** Roy T. M. Sprooten, Gernot G. U. Rohde, Marlou T. H. F. Janssen, Nicolle A. M. Cobben, Emiel F. M. Wouters, Frits M. E. Franssen

**Affiliations:** ^1^ Department of Respiratory Medicine Maastricht University Medical Center Maastricht The Netherlands; ^2^ NUTRIM School of Nutrition and Translational Research in Metabolism Maastricht University Maastricht The Netherlands; ^3^ Department of Respiratory Medicine, Medical Clinic 1 University Hospital Frankfurt Germany; ^4^ CIRO Horn The Netherlands

**Keywords:** Chronic obstructive pulmonary disease, exacerbations, mortality, non‐invasive positive pressure ventilation, response

## Abstract

**Introduction:**

The effectiveness of non‐invasive mechanical ventilation (NIV) in the management of COPD patients suffering from acute respiratory failure (ARF) as a consequence of exacerbation of the disease, is well established. However, data on long‐term outcomes and their predictors, including the individual response to NIV, are scarce.

**Objectives:**

To investigate predictors for short‐ and long‐term mortality in this study population.

**Methods:**

A retrospective cohort study was performed including all patients admitted to the Medium Respiratory Care Unit of Maastricht University Medical Center in Maastricht, the Netherlands, with hospitalized exacerbation of COPD (H‐ECOPD) with ARF requiring NIV for the first time between January 2009 and December 2011. An extensive number of potential predictors of outcomes, including the response to NIV, were determined on admission and during hospitalization. Univariate and multivariate logistic regression was used for statistical analysis.

**Results:**

Seventy‐eight consecutive patients with moderate to severe COPD (mean age 71.0 ± 10.7 years; 48.7% males) were included; In‐hospital, 1‐year and 2‐year mortality rates were 14.1%, 43.6% and 56.4%, respectively. Independent risk factors for 2‐year mortality were: advanced age (odds ratio(OR) 1.025; confidence interval (CI) 1.002‐1.049; *P* = 0.037), prolonged NIV use more than 8 days (OR:1.054;CI:1.006‐1.104; *P* = 0.027) and no successful response to NIV (OR:2.392;CI:1.297‐4.413; *P* = 0.005).

**Conclusion:**

Patients with an H‐ECOPD requiring NIV for the first time, constitute a severely ill patient group with high in‐hospital and 2‐year mortality. This study identified advanced age, NIV use more than 8 days and unsuccessful response to NIV as clinical important independent predictors for long‐term mortality.

**Abbreviations**
ABG: arterial blood gas
ARF: acute respiratory failure
BMI: body mass index
BUN: blood urea nitrogen
CCI: Charlson’s comorbidity index
COPD: chronic obstructive pulmonary disease
DLCO: diffusion capacity
ECOPD: exacerbations of chronic obstructive pulmonary disease
EPAP: expiratory positive airway pressure
ER: emergency room
FEV_1_: forced expiratory volume in one second
FEV_1_/FVC: ratio of post‐bronchodilator forced expiratory volume in 1 second to forced vital capacity
FVC: forced vital capacity
GOLD: global initiative for chronic obstructive lung disease
H‐ECOPD: hospitalized‐ECOPD
ICU: intensive care unit
IMV: invasive mechanical ventilation
IPAP: inspiratory positive airway pressure
LTOT: long‐term oxygen therapy
METC: medical ethical committee
MUMC: Maastricht University Medical Center
NIV: non‐invasive ventilation
RR: respiratory rate
RV: residual volume
S/T mode: spontaneous/timed mode
TLC: total lung capacity

## 1 INTRODUCTION

Severe exacerbations, defined as those requiring hospitalization, are key drivers of health‐care utilization and costs in patients with chronic obstructive pulmonary disease (COPD).^1^ Also, they are associated with accelerated lung function decline^2^ and with poor survival.^3^ While in‐hospital mortality rates vary between 6% and 11%,^4^, ^5^, ^6^, ^7^ 1‐year mortality ranges from 23% to 43%.^5^, ^6^ Advanced age, low body mass index (BMI), chronic hypercapnia and a history of severe exacerbations are well‐recognized independent risk factors for mortality following these events.^8^, ^9^ Also, the presence of cor pulmonale or congestive heart failure^5^ and the long‐term use of oral corticosteroids^6^ are associated with reduced survival following severe exacerbations.^5^

Twenty to thirty percent of patients with severe exacerbations have acute or acute‐on‐chronic hypercapnic respiratory failure,^4^, ^7^ for which non‐invasive mechanical ventilation (NIV) is recommended. NIV reduces the need for intubation, mortality, complications of therapy and length of both hospital stay and intensive care unit (ICU) stay.^10^ However, in‐hospital and short‐term mortality in patients receiving NIV is substantially higher compared to those not requiring ventilatory support, reflecting the increases severity of the exacerbation of COPD (ECOPD).^11^, ^12^, ^13^, ^14^, ^15^ Few studies investigated predictors of mortality in patients receiving NIV.^12^, ^16^, ^17^, ^18^ Older age and low albumin,^16^ and prior domiciliary oxygen use^17^ were identified as the strongest predictors for poor outcome in this population.^16^ However, only a limited number of both patient and exacerbation characteristics as well as details of the NIV intervention were investigated in these studies. Identification of predictors for poor outcome, will aid clinicians and their patients in (shared) decision making regarding the application and duration of continuation of NIV in severe exacerbations with acute or acute‐on‐chronic respiratory failure.

The current study was designed to investigate predictors for short‐ and long‐term mortality in COPD patients requiring NIV for the treatment of acute respiratory failure related to ECOPD for the first time in the course of their disease.

## 2 MATERIALS AND METHODS

A retrospective, observational cohort study was performed at the Respicare, the respiratory medium care unit of the department of respiratory medicine of Maastricht University Medical Center (MUMC) in Maastricht, the Netherlands. Clinical data of patients fulfilling the study criteria between January 1, 2009 and December 31, 2011 were included. Inclusion criteria were: (1) ECOPD, defined as sudden increase in one or more of the following; dyspnea, cough or sputum production and treatment with systemic glucocorticoids and/or antibiotics, (2) confirmation of obstructive lung function by a ratio of post‐bronchodilator forced expiratory volume in 1 second to forced vital capacity (FEV_1_/FVC) <70%^19^ in the medical records of the patient and (3) requiring NIV for the first time assessed by a chest physician according to international guidelines: pH <7.35, PaCO_2_ >6.5kPa, respiratory rate (RR) >23/min).^20^ The index admission was the first hospitalization for acute respiratory failure (ARF) requiring NIV. Besides optimal supportive medical treatment, including maximal bronchodilation, systemic corticosteroids and, in applicable, antibiotics, NIV was initiated in all patients.^19^, ^20^ During NIV treatment, settings were adjusted according to the needs of the patients and guided by blood gas values. Demographic, clinical also including NIV‐related variables and laboratory parameters were retrospectively collected from the electronic records of the patients. The presence of comorbidities was recorded from the medical records according to the following categories: cardiovascular diseases; diabetes mellitus and metabolic diseases; cancers; cognitive and psychological disturbances; renal failure and urogenital tract diseases; infectious diseases and immunological diseases; musculoskeletal diseases and gastro‐intestinal tract diseases. Response to NIV treatment was considered successful if patients fulfilled all of the following criteria: (1) normalization of pH > 7.35, (2) decrease of PaCO_2_ < 6.0 kPa, (3) good tolerance to NIV and 40 no clinical requirement for intubation. During NIV treatment, the tolerance was evaluated with the patient on a daily basis and routinely registered in electronic health records of the patient. NIV treatment was considered prolonged if it was indicated for more than 8 days. In‐hospital mortality was defined as mortality between admission and discharge from the hospital. Mortality during post‐exacerbation inpatient rehabilitation or during stay on an external weaning unit was excluded from this definition. During 2 year follow‐up, data of discharge to home or other residency, all‐cause mortality as well as readmissions were registered. Univariate analyses were performed using the Mann‐Whitney *U* test or *T* test for continuous variables and the Chi square test for categorical data. Cox logistic regression (backward stepwise likelihood ratio) was used for multivariate analyses. A *P* ≤ 0.05 was considered significant. For 2‐year mortality variables that were significant in the univariate analyses or clinically important were entered into the multivariate analyses. Survival was analyzed using the Kaplan‐Meier method. (See supplementary information, Appendix S1‐Material and methods)

## 3 RESULTS

### 3.1 Patient characteristics

**FIGURE 1:**
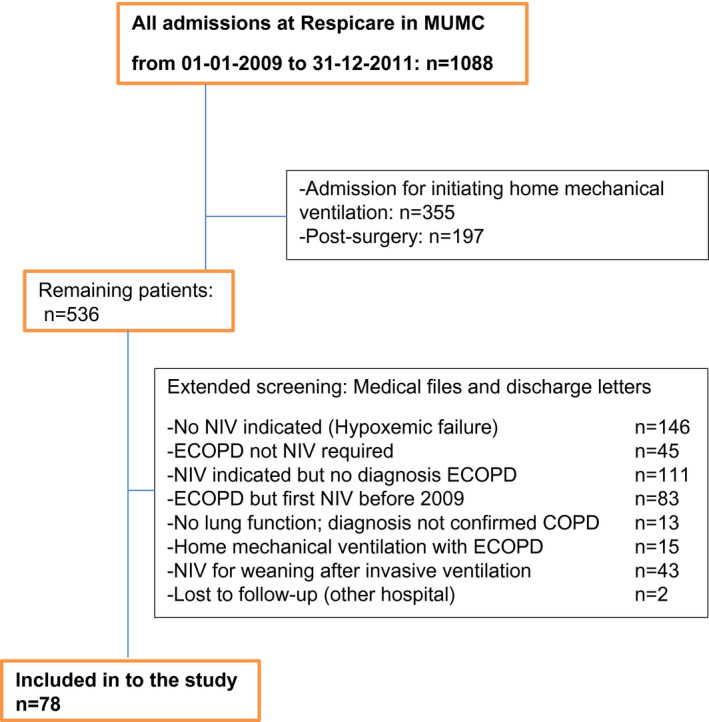
Flowchart of patient selection

Between 2009 and 2011, 1088 patients were admitted to the Respicare Unit. The flow chart for patient selection is depicted in Figure 1. Seventy‐eight COPD patients with severe exacerbation requiring firstly NIV for ARF were included into our analysis. The clinical characteristics of the study population are shown in Tables 1 and 2 and detailed information can be find in the supplemental data (Tables S1‐S4).

**TABLE 1:** Characteristics of patients hospitalized for ECOPD with acute respiratory failure and requiring non‐invasive ventilation at first, stratified by in‐hospital and long‐term survival

	In‐hospital survivors (n = 67)	In‐hospital deaths (n = 11)	Long‐term survivors (n = 34)	Long‐term deaths (n = 44)
Male gender	30 (44.8)	8 (72.7)	11 (28.9)*	27 (71.1)*
Age at admission, years	69.9 ± 10.8*	77.5 ± 7.3*	66.9 ± 11.6*	74.1 ± 8.9*
Body mass index, kg/m^2^	23.9 ± 5.4*	20.6 ± 2.2*	24.8 ± 5.6	22.3 ± 4.7
Pack years (n = 32/29/3)	42.5 ± 12.1	53.3 ± 11.5	39.2 ± 12.4*	48.9 ± 10.2*
Post‐BD FEV_1_, %pred	40.1 (28.8‐53.6)	36.1 (27.8‐44.5)	40.2 (29.9‐54.3)	36.9 (27.2‐49.8)
Frequent exacerbators (>1)	9 (13.4)	2 (18.2)	4 (11.8)	7 (15.9)
CCI	2.0 (1‐3)*	3.0 (2‐4)*	2 (1‐3)	2 (1‐3)

Note

Categorical data are presented as n (%), non‐parametric data as median (IQR) and parametric data as mean ± (SD). Chi‐X^2^, Kruskal‐wallis test and *T* test were used for statistical analysis, respectively.

Abbrevations: CCI, Charlson Comorbidity Index; FEV1, forced expiratory volume in the first second; Post‐BD, post bronchodilator.

* *P*‐value < 0.05;

^#^ *P*‐value < 0.001.

**TABLE 2:** Non‐invasive ventilation (NIV) results of patients hospitalized for ECOPD with acute respiratory failure and requiring NIV at first, stratified by in‐hospital and long‐term survival

	In‐hospital survivors (n = 67)	In‐hospital deaths (n = 11)	Long‐term survivors (n = 34)	Long‐term deaths (n = 44)
ABG before start NIV(n = 77)				
pH	7.28 (7.22‐7.31)*	7.24 (7.1‐7.26)*	7.28 (7.23‐7.31)*	7.25 (7.18‐7.29)*
<7.25	21 (31.8)*	7 (63.6)*	8 (23.5)*	20 (46.5)*
7.25‐7.35	45 (68.2)*	4 (36.4)*	26 (76.5)*	23 (53.5)*
PaCO_2_, kPa	10.0 (8.5‐11.0)	10.8 (8.3‐12.1)	9.7 (8.5‐10.5)	10.3 (8.6‐11.5)
HCO_3_^−^, mmol/L	31.9 (28.0‐36.2)*	26.7 (25.4‐36.8)*	31.4 (27.8‐73.0)	31.8 (26.7‐36.0)
Base excess	3.7 (−0.3‐6.6)	2.9 (−5.4‐6.6)	3.9 (−0.3‐7.0)	3.1 (−2.6‐6.5)
<−2.5	9 (13.6)*	5 (45.5)*	3 (8.8)	11 (25.6)
−2.5‐2.5	21 (31.8)*	0*	12 (35.3)	9 (20.9)
>2.5	36 (54.5)*	6 (54.5)*	19 (55.9)	23 (53.5)
Clinical data				
LOS, days	17.0 (11.0‐27.0)	11.0 (3.0‐31.0)	13.5 (10.0‐18.3)	17.0 (8.3‐28.0)
NIV, days	5.0 (2.0‐7.0)	7.0 (3.0‐11.0)	4.0 (3.0‐6.0)	5.0 (2.0‐10.8)
NIV >8 days	11 (16.4)	4 (36.4)	3 (8.8)*	12 (27.3)*
IPAP, cm H_2_O	20.5 ± 4.3	22.9 ± 3.8	21.2 ± 3.9	20.6 ± 4.5
EPAP, cm H_2_O	6.2 ± 1.5	7.1 ± 1.4	6.3 ± 1.3	6.4 ± 1.7
NIV response after 1 hours				
Delta pCO_2_ before NIV‐1h, kPa	1.4 ± 1.3	1.6 ± 1.1	1.1 ± 1.1	1.6 ± 1.3
pH ≥ 7.35 at 1 hours	21 (31.8)	1 (9.1)	12 (35.3)	10 (23.3)
RR at 1 h, /min	22.2 ± 5.9*	26.7 ± 4.4*	21.0 ± 6.1*	24.3 ± 5.4*
RR >20/min at 1 hours	31 (57.4)	8 (88.9)	14 (48.3)*	25 (73.5)*

Note

Categorical data are presented as n(%), non‐parametric data as median (IQR) and parametric data as mean ± (SD). Chi‐X^2^, Kruskal‐wallis test and *T* test were used for statistical analysis, respectively.

Abbrevations: ABG, arterial blood gas; BE, base excess; EPAP, Expiratory positive air pressure; IPAP inspiratory positive air pressure; LOS, Length of hospital stay; NIV, non‐invasive ventilation; RR, respiratory rate.

* *P*‐value < 0.05;

^#^ *P*‐value < 0.001.

The median length of hospital stay was 16.5 (11.0‐28.3) days. After discharge, 49 patients went home, 16 were admitted to a rehabilitation center or to a nursery home. Four patients went home with palliative care. Four patients received directly after admission home mechanical ventilation as non‐invasive ventilation, whereas is 11 (14.1) patients long‐term oxygen therapy was initiated. The readmission rate was, respectively, 20.8% at 90 days, 39.8% at 1 year and 41% after 2 years of follow‐up. Data in detail about readmissions are shown in the supplemental data (Table S5).

### 3.2 Predictors of mortality

**FIGURE 2:**
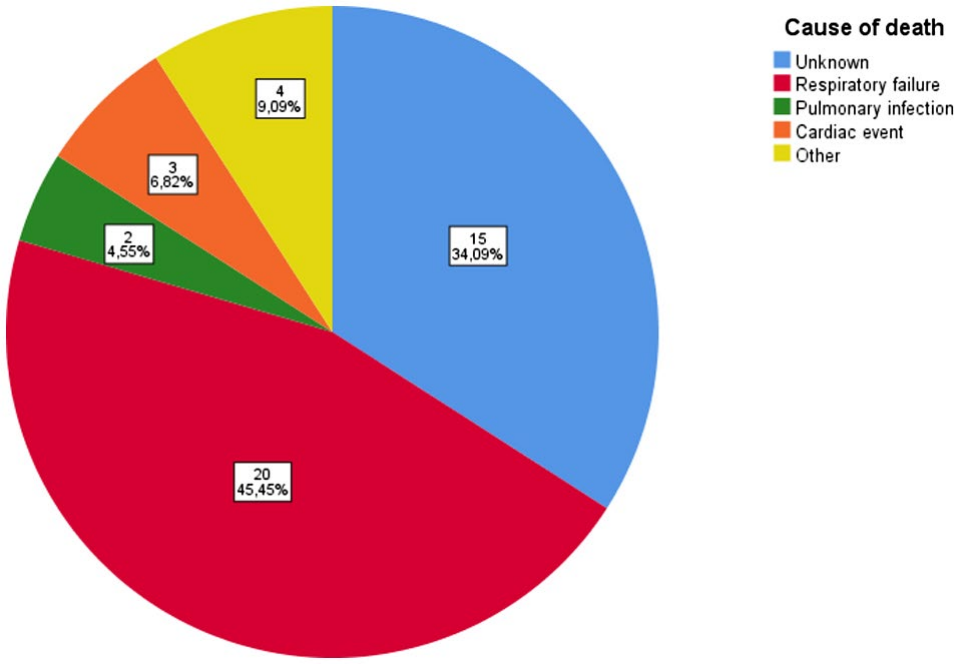
Cause of death during 2‐year follow‐up in patients cohort hospitalized for ECOPD with acute respiratory failure and requiring non‐invasive ventilation at first

In‐hospital mortality was 14.1% (n = 11). Mortality rate during follow‐up was, respectively, 29.5% at 90 days, 43.6% at 1 year and 56.4% at 2 years. Most of the deaths died because of respiratory failure due to ECOPD (see Figure 2).

Results of the univariate comparison between the in‐hospital survivors and non‐survivors are shown in Tables 1 and 2. Significant predictors are: advanced age at admission, smoking status, prednisone maintenance therapy, high CCI especially gastro‐intestinal tract involvement, low BMI, Blood urea nitrogen (BUN) ≥ 8mmol/L^4^, high troponin levels (n = 38), lower pH before start NIV, bicarbonate ≥ 27.0 mmol/L, changed limited treatment restriction at admission. After 1 hour of NIV, RR was significant higher in deceased patients. Moreover, the pH and PaCO_2_ and the changes between the time points 1, 24 and 72 hours did not show any significant differences between the groups. In 43 (55.1%) patients NIV was assessed as successful. NIV was successful in 3 out of 11 in‐hospital non‐survivors compared to 40 out of 67 survivors (27.3% vs 59.7%, *P* = 0.045 (pearson Ch‐Square). The survivor group had a significantly better tolerance to NIV compared to the non‐survivors (80.6% vs 12.5%, *P* = 0.000 (pearson Chi‐square)). There was no significant difference in the percentage of patients with successful response between non‐survivors and survivors after 2 years follow‐up (see supplementary information Table S4).

The univariate comparison between the survivors and non‐survivors after 2‐year follow‐up are presented in Tables 1 and 2. Prior to NIV, several significant negative predictors were found: male gender, higher amount of pack years, prednisone maintenance therapy, atrial fibrillation, low pH and change in medical policy restrictions. RR after 1 hour use of NIV was significantly higher in the non‐survivors. The delta PaCO_2_ before start and at termination NIV was different but failed to reach statistical significance (*P* = 0.058).

**FIGURE 3:**
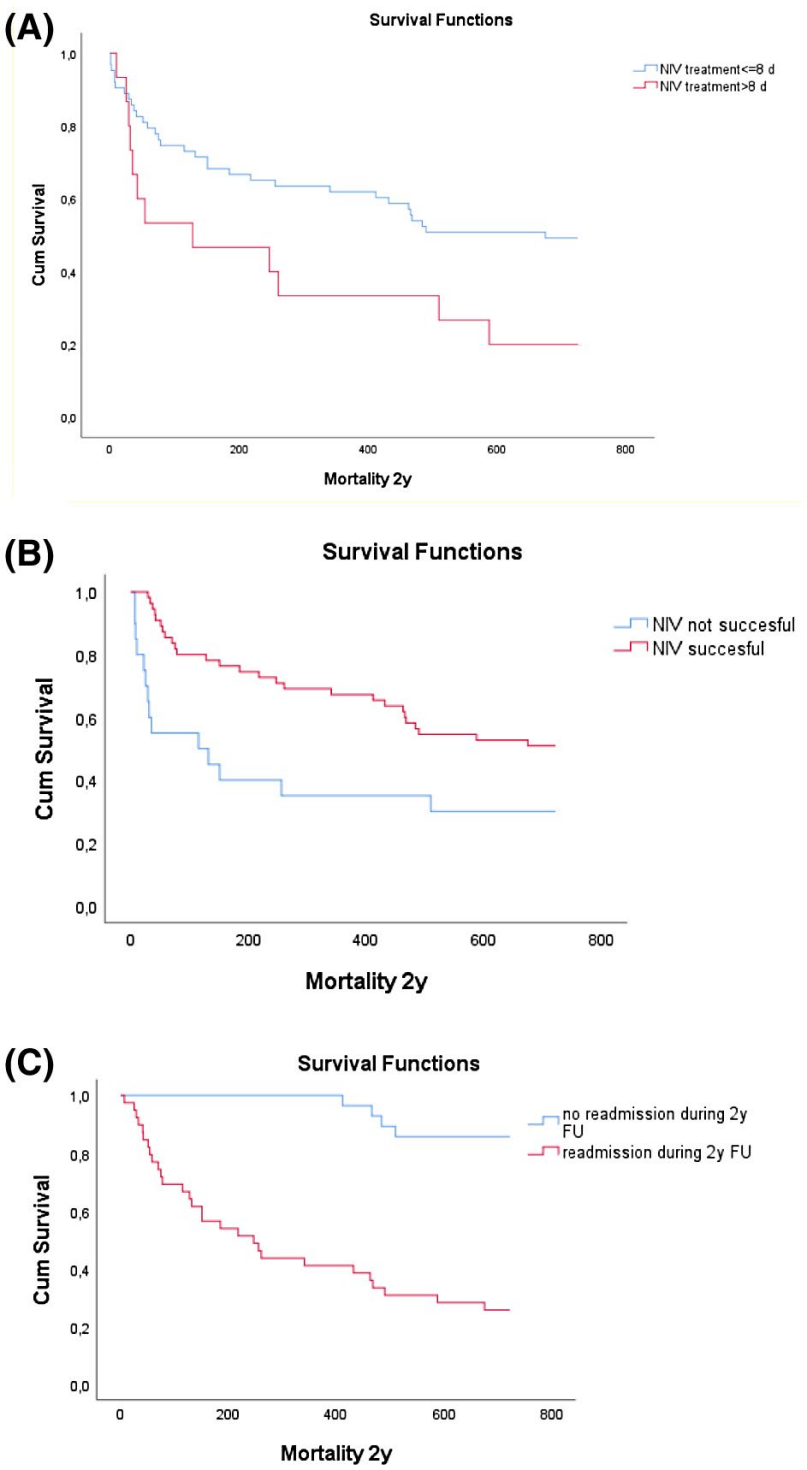
Overall mortality after 2 years follow‐up of patients hospitalized for ECOPD with acute respiratory failure and requiring non‐invasive ventilation at first, stratified by, respectively, days treated with NIV (>8 days or ≤8 days), see figure A, (n = 78; Logrank 0.025, Breslow 0.040, Pearson Chi‐X^2^ 0.040); successful NIV (home mechanical ventilation is regarded as successful) (yes or no), see figure B, (n = 78; Log rank 0.011, Breslow 0.002) and readmission during follow‐up, see figure C, (n = 67; Log rank 0.0001, Breslow 0.0001). d, days

In the multivariate analysis were entered the following clinically relevant or significant univariate analysis variables: advanced age, gender, pH before start NIV, RR >20 minutes, delta paCO_2_ before start NIV versus at termination NIV, duration NIV (days), response (tolerance) to NIV. As independent factors associated with 2‐year mortality advanced age, prolonged (>8 days) NIV use and the response to NIV assessed to be unsuccessful were identified (Table 3). Figure 3 show the Kaplan Meier survival curve of prolonged (>8 days) NIV use (Logrank 0.025; Breslow 0.040), unsuccessful NIV (Logrank 0.011; Breslow 0.002) and occurrence of readmission after discharge (Logrank 0.0001; Breslow 0.0001), which have negative impact on survival. (See supplementary information, Appendix S2‐Results)

**TABLE 3:** Multivariate model using seven coefficients to analyse 2 years mortality in patients hospitalized for ECOPD with acute respiratory failure and requiring non‐invasive ventilation at first. Shown in table is the predicted odds ratio (OR) based on the regression coefficient Beta, the 95% confidence interval for Beta and the uncorrected p‐value using the backward stepwise cox regression

	OR	95% CI for Exp(B)	*P*‐value
Age* (years)	1.025	1.002‐1.049	0.037
Gender^#^ (female = 0)	0.808	0.437‐1.382	0.436
pH before start NIV*	0.136	0.002‐11.478	0.378
RR >20/min^#^ (>20/min = 0)	0.945	0.528‐1.692	0.850
Delta PaCO_2_ before NIV‐ stop NIV (kPa)*	1.000	0.972‐1.029	0.986
Total days NIV* (days)	1.054	1.006‐1.104	0.027
Response tolerance/ adherence NIV^#^ (not successful = 0)	2.392	1.297‐4.413	0.005

Abbreviations: CI, confidence interval; kPa, kilopascal; NIV, non‐invasive ventilation; OR, odds ratio; RR, respiratory rate.

*Note:* All data available in 60 subjects.

* Continues variable.

^#^ Categorical variable.

## 4 DISCUSSION

This study investigated the demographic, biochemical, clinical and intervention‐related predictors of short‐ and long‐term mortality in patients requiring non‐invasive positive pressure ventilation for the treatment of acute respiratory failure. Fourteen percent of patients died during hospital stay, while 56% died within 2 years. Older age, NIV use more than 8 days and non‐successful response to NIV were identified as independent predictors for long‐term mortality.

There is a large variation in the reported in‐hospital mortality rates for patients with severe exacerbations and need for NIV. Differences in study population, severity of ECOPD and clinical setting contribute to this variation. Some studies reported low mortality rates of 4‐5%.^13^, ^16^, ^21^ A large nationwide study in the United States of America published by Chandra et al. found a mortality rate of 9% in patients with ECOPD and ARF who only needed NIV.^11^ Plant et al, one of the first randomized trials in COPD and NIV, reported an in‐hospital mortality of 10% in the NIV group.^22^ Another study in patients managed in intermediate care unit reported a mortality rate of 20%.^23^ Sainaghi et al reported mortality rates of patients admitted with ECOPD and ARF requiring NIV managed in‐ward as high as 27%.^24^ Patients with ECOPD and ARF started with NIV and transitioned to invasive mechanical ventilation (IMV) had a worse mortality rate up to 27%.^11^ In a prospective study mortality rate was studied in patients admitted to respiratory intensive care unit with ECOPD and acute hypercapnic respiratory failure. From the 151 enrolled patients 87 (57.6%) received mechanical ventilation and 22 patients (14.6%) NIV. The in‐hospital mortality was 33.1%.^25^ The authors attributed the higher mortality rate to higher severity of illness and higher intubation rates.

In our study the in‐hospital mortality rate is 14.1% which is higher than several studies reported.^13^, ^16^, ^21^, ^26^ This difference can be explained by the severity of ARF, reflected in lower pH levels in our study population and the severity of illness. Still the in‐hospital mortality we found in our study is much lower in patients with hospitalized‐ECOPD (H‐ECOPD) and ARF intubated and required IMV (25%).^27^

There is limited data about long‐term mortality in patients admitted for ECOPD with acute respiratory failure requiring NIV and results are again variable. In a randomized controlled study by Plant et al the 1‐year survival in the NIV group was 61.6%.^22^ A retrospective single center study in Spain reported a long‐term mortality at 1, 2 and 3 years, respectively, as 30%, 47.7% and 74%.^16^ Chung et al investigated the long‐term mortality in patients admitted for ECOPD with acute respiratory failure first requiring NIV and found a 1‐year mortality of 28%, a 2‐year mortality of 48% and a 5‐year mortality of 74%.^17^ Another study published by Titlestad showed comparable outcomes.^12^ Our study revealed a 2‐year mortality rate of 56.4%. The slightly higher mortality rates at 1 and 2 years in our study compared to the study from Chung et al can be explained by the fact that Chung only included patients who survived the initial hospital admission whereas in our study we analyzed all patients admitted.

Identifying factors influencing in‐hospital and long‐term mortality is pivotal in management of these severely ill patients, as NIV may be stressful and demanding and adverse events may occur. Therefore, initiation and continuation of NIV treatment is not only a decision by the pulmonologist, but also a moment of shared‐decision making with the patient and his relatives. This discussion benefits from well‐established predictors for short‐ and long‐term outcomes. The present study observed that patients with advanced age had worse outcome, which is in concordance with previous studies.^4^, ^5^, ^6^, ^9^, ^12^, ^15^, ^17^, ^26^, ^28^, ^29^

Another independent predictor for 2‐year mortality was prolonged use of NIV, especially longer than 8 days. This finding is comparable with findings of a study published by Penuelas.^30^ They found in a cohort of mechanical ventilated patients that prolonged weaning of a duration longer than 7 days is associated with an increased risk for death. However, these patients underwent IMV and not NIV.

The present study observed that response to NIV considered as unsuccessful was found a negative independent predictor for long‐term mortality. Literature investigating the meaning of the response of NIV and its impact on long‐term survival after H‐ECOPD is scarce. It has been reported that the more seriously ill patients, as well as those with a poorer initial response to NIV are more likely to suffer NIV failure.^31^, ^32^ Previous studies revealed several factors predicting NIV failure (short‐term) which are severity hypercapnia and acidosis at admission, failure in improvement first hour, diaphragmatic dysfunction, patient ventilator asynchrony, poor nutritional status and pneumonia as a cause of ARF.^13^, ^21^, ^31^, ^33^, ^34^, ^35^ However, most studies about this subject were done in ICU populations. Plant et al demonstrated that the severity of acidosis, degree of hypercapnia, severity of hypoxia were associated with failure of NIV treatment and subsequent negative impact on mortality, while decrease in respiratory rate after 4 hours and improvement of pH were associated with successful outcome.^22^ Akyil found in their study higher white blood cell count, lower hematocrit, lower levels of albumin at admission, lower pH and higher PaCO_2_ level at 24 hours as predictors for negative outcome.^16^ In a more real life setting, Nicolini et al. demonstrated that patients with fewer comorbidities, less severe illness and improvement in ABG parameters within 1 hour are more likely to have successful outcome.^21^ Furthermore, the appropriate choice of ventilator modality and interface and the level of team experience and patient’s cooperation play a pivotal role in successful NIV treatment.^36^, ^37^, ^38^ These findings are in line with our study. Few studies reported factors that have negative impact on long‐term survival. Titlestad et al identified advanced age and do‐not‐intubate orders as an independent negative predictor.^12^ Levels of pH and PaCO_2_ were not identified as a predictor. Another study described advanced age, lower BMI and LTOT as long‐term mortality predictors. Again this study found that ABG levels at admission, 2 hours after NIV initiation and at discharge were correlated with survival. These finding are in line with a recently published study by Steriade et al in which 89 patient with ECOPD requiring NIV were included. They reported that the mortality and NIV failure (defined as intubation or in‐hospital death) was not influenced by the arterial blood gas values in the first 6 hours of NIV. Unfortunately, the data are not sufficient to analyze the impact on long‐term mortality.^26^

In the current study prednisone maintenance therapy was identified as a predictor of in‐hospital and long‐term mortality in the univariate analysis. This could be due to adverse effects of prednisone or confounding with underlying disease severity. Our results confirm the findings by Groenewegen et al, who reported that the use of oral corticosteroids was an independent risk factor for mortality after H‐ECOPD.^6^

Significant findings in the univariate analysis for potential predictors of 2‐year outcome were male gender, advanced age at admission, prednisone maintenance. A lower pH before start of NIV was also a significant predictor. Response to NIV according normalizing pH or lowering PaCO_2_ did not seek out as a significant predictor. Regarding male gender, which in our study was a predictor for mortality, several reports are published.^4^, ^12^ Slenter et al also showed a prominent association of male gender with an increased risk of death and indicated that male COPD patients admitted to the hospital with ECOPD had a worse health status.^4^ Another study performed in patients with ECOPD and ARF requiring NIV observed a trend towards better long‐term (5 years) survival in females compared to males.^12^ The explanation of this observation could be the historical fact that males might have had a higher exposure of noxious particles (cigarette smoke or occupational lung irritants) contributing to worse long‐term survival.^39^ However, our data cannot support this theory.

The need of repeated hospitalizations for ECOPD is associated with poor outcomes. In our study the readmission rate in the first year was almost 40%. Chung et al reported a readmission rate in the first year of follow‐up of 56%.^17^ This stresses the importance of post hospitalization care in this very frail patient group.

### 4.1 Strengths and limitations

The thorough assessment of patient demographics, clinical, biometrical and biochemical parameters and recording of details of the NIV intervention in a real‐life clinical setting with long‐term follow‐up is the strength of the current study. Also, the collected data was highly complete with few missing patients and no loss to follow‐up. The diagnosis COPD was well documented and confirmed by spirometry. Furthermore, to our knowledge this is the first study that investigated the short and long‐term mortality related to the response to or successful use of NIV.

Our results should be interpreted in light of some limitations. First, its retrospective design could lead to information bias. The presented study was performed in a single center which limits the generalizability of our results. Moreover, potential important prognostic factors such as the SAPS II score,^21^ patient’s activities of daily living, dyspnea score^40^ could were not obtained, because they were not available. The NIV tolerance was subjectively assessed clinically by the nurses and pulmonologists. The sample size of 78 patients is relatively small and may have limited the detection of other important predictors.

### 4.2 Clinical implications and summary

This retrospective, single‐center study shows that patients who are admitted to the hospital for a first hospitalization requiring NIV for ARF in an ECOPD, are a severely ill patient group with high short and long‐term mortality rates. Not the response of levels of PaCO_2_ or pH but older age, NIV use more than 8 days and lack of successful NIV response are independent prognostic factors for 2‐year mortality. These results could help clinicians to predict the prognosis in these patients and facilitate “shared decision making” with these severe ill patients. Also, continuation of NIV in patients above 8 days can be debated. Obviously, further research is needed to determine the exact value of these factors to predict the prognosis in patients admitted with acute respiratory failure requiring NIV at first and its response to NIV.

## CONFLICT OF INTEREST

The authors reported their conflict of interest in the enclosed ICMJE form for Disclosure of Potential Conflicts (forms are uploaded).

## AUTHOR CONTRIBUTIONS

*Designed the study in corporation with the co‐authors*: Sprooten

*Drafted the manuscript*: Sprooten

*Collected the data*: Janssen

*Performed the statistical analysis*: Sprooten

*Wrote the paper*: Sprooten

All authors read and approved the final manuscript.

## ETHICS

The study protocol was reviewed and approved by the Medical Ethics Committee (MUMC, METC 14‐04‐008) and conducted according to the Declaration of Helsinki (59^nd^ WMA General Assembly, Seoul, October 2008) and Good Clinical Practice guidelines.

## Supporting information

Supplementary Material
