# Supplementary material for: Predictors for long‐term mortality in COPD patients requiring non‐invasive positive pressure ventilation for the treatment of acute respiratory failure
Source: Clin Respir J. 2020 Sep 22;14(12):1144–52. doi: 10.1111/crj.13251 (PMC7756413; doi:10.1111/crj.13251)
Supplement: Supplementary file 1 — Supplementary Material [file CRJ-14-1144-s001.doc]

**SUPPLEMENTARY INFORMATION**

**TITLE: Predictors for long-term mortality in COPD patients requiring non-invasive positive pressure ventilation for the treatment of acute respiratory failure**

**Authors’ names:**

R.T.M. Sprooten^1,2^, G.G.U. Rohde^4^, M.T.H.F. Janssen^1^, N.A.M. Cobben^1^, E.F.M. Wouters^1,2,3^, F.M.E. Franssen^1,2,3^

**Authors’ affiliation(s):**

^1^ Department of Respiratory Medicine, Maastricht University Medical Center, Maastricht, The Netherlands

^2^ NUTRIM School of Nutrition and Translational Research in Metabolism, Maastricht University, Maastricht, The Netherlands

^3^ CIRO, Horn, The Netherlands

^4^ Department of Respiratory Medicine, Medical Clinic 1, University Hospital Frankfurt, Germany

**Appendix S1-Material and methods**

**Material and Methods (extended version)**

*Design and population*

A retrospective, observational cohort study was performed at the Respicare, the respiratory medium care unit of the department of respiratory medicine of Maastricht University Medical Center (MUMC) in Maastricht, the Netherlands. Clinical data of patients fulfilling the study criteria between January 1, 2009 and December 31, 2011 were included. Inclusion criteria were: 1) ECOPD, defined as sudden increase in one or more of the following; dyspnea, cough or sputum production and treatment with systemic glucocorticoids and/or antibiotics, 2) confirmation of obstructive lung function by a ratio of post-bronchodilator forced expiratory volume in 1 second to forced vital capacity (FEV_1_/FVC) <70% [1] in the medical records of the patient and 3) requiring NIV for the first time assessed by a chest physician according to international guidelines: pH <7.35, PaCO_2_ >6.5kPa, respiratory rate (RR) >23/min) [2]. Exclusion criteria were: 1) age <40 years, 2) active pulmonary malignancy based on medical records, 3) follow-up in another hospital and 4) primary reason for hospitalization other than exacerbation. The index admission was the first hospitalization for acute respiratory failure (ARF) requiring NIV. The study protocol was reviewed and approved by the Medical Ethics Committee (MUMC, METC 14-04-008) and conducted according to the Declaration of Helsinki (59^nd^ WMA General Assembly, Seoul, October 2008) and Good Clinical Practice guidelines.

Besides optimal supportive medical treatment, including maximal bronchodilation, systemic corticosteroids and, in applicable, antibiotics, NIV was initiated in all patients [1, 2]. The initial ventilator settings (BIPAP vision, ventilator, Philips, Respironics, Netherlands, Eindhoven) were spontaneous/timed mode (S/T mode), inspiratory positive airway pressure (IPAP) to expiratory positive airway pressure (EPAP) ratio 14/4 cmH_2_O, backup frequency 12/min, inspiratory time 1.0s, rise time 50ms. During NIV treatment, settings were adjusted according to the needs of the patients and guided by blood gas values. After stabilization of the patient, weaning was started.

*Data collection*

Demographic, clinical and laboratory parameters were retrospectively collected from the electronic records of the patients. The following variables were recorded: pre-admission data such as demographics, smoking and medical history including comorbidities, previous admissions for severe exacerbations, post-bronchodilator FEV_1_ and FVC, FEV_1_/FVC ratio, GOLD stage for airflow limitation, residual volume (RV), total lung capacity (TLC), diffusion capacity (DLCO), need for home care, domestic situation, respiratory medications, use of long term oxygen treatment (LTOT) and arterial blood gases (ABG) before hospitalization. Comorbidities were quantified using the Charlson’s comorbidity index (CCI) [3]. The presence of comorbidities was recorded from the medical records according to the following categories: cardiovascular diseases; diabetes mellitus and metabolic diseases; cancers, cognitive and psychological disturbances; renal failure and urogenital tract diseases; infectious diseases and immunological diseases; musculoskeletal diseases and gastro-intestinal tract diseases. On admission and during treatment, the following data were collected: vital signs, physical examination, BMI, ABG, blood chemistry, electrocardiogram (rhythm and signs of ischemia), chest X-ray (consolidation and signs of congestion), ABG before start of NIV and after 1, 24 and 72 hours of NIV and before stop. Also, length of hospital stay was recorded. NIV related variables were collected: duration of NIV, ventilator settings such as IPAP, EPAP, reason to discontinue NIV, patient cooperation and tolerance of NIV, complications and the need for intubation. The clinical response to NIV was assessed at 1 hour, 24 and 72 hours after start NIV. Response to NIV treatment was considered successful if patients fulfilled all of the following criteria: 1) normalization of pH > 7.35, 2) decrease of PaCO2 < 6.0kPa, 3) good tolerance to NIV and 4) no clinical requirement for intubation. During NIV treatment, the tolerance of NIV was evaluated with the patient on a daily basis and routinely registered in electronic health records of the patient. NIV treatment was considered prolonged if it was indicated for more than 8 days. In-hospital mortality was defined as mortality between admission and discharge from the hospital. Mortality during post-exacerbation inpatient rehabilitation or during stay on an external weaning unit was excluded from this definition. During two year follow-up, data of discharge to home or other residency, all-cause mortality as well as readmissions were registered. In case of an in-hospital death the date was recorded and verified. The date of death after hospital discharge was verified from hospital records, general practitioners records or death records of the patients’ residence.

*Statistical analysis*

SPSS version 23 software was used for all statistical analyses. First, descriptive data analysis was performed. The distribution of data was assessed by Kolmogorov-Smirnov test. Normally distributed data are reported as mean (standard deviation) whereas non-parametric data are expressed as median (interquartile range). Categorical data are presented as percentages (n(%)). Univariate analyses were performed using the Mann-Whitney U test or T-test for continuous variables and the Chi square test for categorical data. All variables were included into the univariate analyses. Cox logistic regression (backward stepwise likelihood ratio) was used for multivariate analyses. A p ≤0.05 was considered significant. For 2-year mortality variables that were significant in the univariate analyses or clinically important were entered into the multivariate analyses. Survival was analyzed using the Kaplan-Meier method.

**Appendix S2-Results**

**Results (extended version)**

*Patient characteristics*

Between 2009 and 2011, 1088 patients were admitted to the Respicare Unit. The flow chart for patient selection is depicted in figure 1. Following the in- and exclusion criteria of this study, 78 COPD patients with severe exacerbation requiring NIV for ARF were included into our analysis. The clinical characteristics of the study population are shown in tables 1-4 (see supplemental data online). Patients were elderly males and females with moderate to very severe COPD. Almost half of all patients had been hospitalized for ECOPD in the previous 2 years and more than half were current smokers. The majority 55 (73.3%) of patients received additional professional support at home. Thirty patients (38.4%) had LTOT and comorbidities were common. The majority of patients with previous blood gas results were hypercapnic with normal pH at that time (table 1 and 3 (see supplemental data online)). Vital signs and laboratory results at the time of presentation at the emergency room (ER) are presented in table 2 and 4 (see supplemental data online). The median respiratory rate was 30 (24-34)/min, the median SpO_2_ was 90% (82-94%). A third of the patients had underweight, 36.4% had a pH <7.25, the majority had a PaCO_2_ >9.1kPa. In almost 20% treatment restrictions were present before admission; in almost half of patients restrictions were discussed and changed on ER. In 26 patients (32.1%) the chest X-ray showed a consolidation at admission, in 10 patients (12.3%) a pleural effusion and in 14 patients (17.3%) signs of heart failure.

*Hospital admission, discharge and long term follow-up*

The median length of hospital stay was 16.5 (11.0-28.3) days. After discharge, 49 patients went home, 16 were admitted to a rehabilitation center or to a nursery home. Four patients went home with palliative care. Four patients received directly after admission home mechanical ventilation as non-invasive ventilation, whereas is 11 (14.1) patients long-term oxygen therapy was initiated. Twelve patients (15.4%) were admitted to post-exacerbation pulmonary rehabilitation. The readmission rate was respectively 20.8% at 90 days, 39.8% at one year and 41% after 2 years of follow-up. Data in detail about readmissions are shown in the supplemental data (table 5).

Predictors of mortality

In-hospital mortality was 14,1%(n=11). Mortality rate during follow-up was respectively, 29.5% at 90 days, 43.6% at one year and 56.4% at two years. Most of the deaths died because of respiratory failure due to ECOPD (see figure 2 in the main manuscript).

Results of the univariate comparison between the in-hospital survivors and non-survivors are shown in tables 1-2 (see supplemental data online). Significant predictors are: advanced age at admission, smoking status, prednisone maintenance therapy, high CCI especially gastro-intestinal tract involvement, low BMI, Blood urea nitrogen (BUN) ≥8mmol/L[4], high troponin levels (n=38), lower pH before start NIV, bicarbonate ≥27.0 mmol/L, changed limited treatment restriction at admission. After one hour of NIV, RR was significant higher in deceased patients. Moreover, the pH and PaCO_2_ and the changes between the time points 1, 24 and 72 hours did not show any significant differences between the groups. In 43 (55.1%) patients NIV was assessed as successful. NIV was successful in 3 out of 11 in-hospital non-survivors compared to 40 out of 67 survivors (27.3% vs 59.7%, p=0.045 (pearson Ch-Square). The survivor group had a significantly better tolerance to NIV compared to the non-survivors (80.6% vs 12.5%, p=0.000 (pearson Chi-square)). There was no significant difference in the percentage of patients with successful response between non-survivors and survivors after two years follow up (see table 4 (see supplemental data online)).

The univariate comparison between the survivors and non-survivors after 2-year follow-up are presented in tables 3 and 4. Prior to NIV, several significant negative predictors were found: male gender, higher amount of pack years, prednisone maintenance therapy, atrial fibrillation, low pH, change in medical policy restrictions. RR after 1 hour use of NIV was significantly higher in the non-survivors. The delta PaCO_2_ before start and at termination NIV was different but failed to reach statistical significance (p=0.058).

In the multivariate analysis were entered the following clinically relevant or significant univariate analysis variables: advanced age, gender, pH before start NIV, RR >20min, delta paCO_2_ before start NIV vs at termination NIV, duration NIV (days), response (tolerance) to NIV. As independent factors associated with 2-year mortality advanced age, prolonged (>8 days) NIV use and the response (successful or not successful) to NIV assessed to be unsuccessful were identified (table 6) (see main manuscript). Figure 3 (see main manuscript) show the Kaplan Meier survival curve of prolonged (>8 days) NIV use (Logrank 0.025; Breslow 0.040) and unsuccessful NIV (Logrank 0.011; Breslow 0.002), and occurrence of readmission after discharge (Logrank 0.0001; Breslow 0.0001), which have negative impact on survival.

**Tables and Figures (extended version)**

**Table 1.** Characteristics of patients hospitalized for ECOPD with acute respiratory failure and requiring non-invasive ventilation at first, stratified by in-hospital mortality.

|  | **All**  **(n=78)** | **Hospital survivors**  **(n=67)** | **Hospital deaths**  **(n=11)** | **p-value** |  |
| --- | --- | --- | --- | --- | --- |
| **Male gender** | 38 (48.7) | 30 (44.8) | 8 (72.7) | *0.086* |  |
| **Age at admission, y** | 71.0±10.7 | 69.9±10.8 | 77.5±7.3 | *0.027* |  |
| ***Smoking status (n=74)*** |  |  |  |  |  |
| ***Non smoker*** | *1 (1.3)* | *0* | *1 (11.1)* | *0.001* |  |
| ***Former smoker*** | *32 (41.0)* | *25 (38.5)* | *7 (77.8)* |  |  |
| ***Current smoker*** | *41 (52.6)* | *40 (61.5)* | *1 (11.1)* |  |  |
| **Pack years (n=32/29/3)** | 43.5±12.3 | 42.5±12.1 | 53.3±11.5 | *0.148* |  |
| ***GOLD stage*** |  |  |  |  |  |
| ***Gold II*** | *17 (21.8)* | *8 (23.5)* | *8 (18.2)* |  |  |
| ***Gold III*** | *21 (26.9)* | *9 (26.5)* | *12 (27.3)* |  |  |
| ***Gold IV*** | *40 (51.3)* | *16 (47.1)* | *24 (54.5)* |  |  |
| ***Post-BD* FEV_1_, %pred** | 39.0(28.6-52.9) | 40.1(28.8-53.6) | 36.1(27.8-44.5) | *0.438* |  |
| ***Post-BD* *FEV_1_, L*** | *0.9±0.5* | *1.0±0.5* | *0.9±0.26* | *0.455* |  |
| ***FEV_1_/FVC*** | *38.3±11.2* | *38.7±16.0* | *35.4±9.3* | *0.355* |  |
| ***RV/TLC, % (n=53/46/7)*** | *61.9±10.0* | *61.6±10.5* | *64.0±5.8* | *0.568* |  |
| ***TLCO/VA, %pred (n=51/44/7)*** | *51.1±21.8* | *51.2±22.6* | *50.3±16.6* | *0.927* |  |
| ***Hospitalization for ECOPD previous year*** | *26 (33.3)* | *23 (34.3)* | *3 (27.3)* | *0.645* |  |
| ***0-1***  **Frequent exacerbators (>1)** | 67 (85.9)  11 (14.1) | *58 (86.6)*  9 (13.4) | *9 (81.8)*  2 (18.2) | *0.675* |  |
| ***Living status (n=75)***  ***At home***  ***Alone***  ***Alone with care***  ***Cohabitation***  ***Cohabitation with care*** | *65 (83.3)*  *10 (12.8)*  *20 (25.6)*  *10 (12.8)*  *25 (32.1)* | *56 (86.2)*  *10 (14.9)*  *18 (26.9)*  *9 (13.4)*  *19 (28.4)* | *10 (90.9)*  *0*  *2 (20.0)*  *1 (10.0)*  *6 (60.0)* | *0.666*  *0.121* |  |
| ***Resident (not at home)*** | *10 (14.1)* | *9 (13.8)* | *1 (9.1)* | *0.666* |  |
| ***No additional care*** | *20 (25.6)* | *19 (29.2)* | *1 (10.0)* | *0.200* |  |
| ***Treatment for COPD yes*** | *73 (93.6)* | *62 (92.5)* | *11 (100)* | *0.645* |  |
| ***SABA*** | *43 (55.1)* | *37 (55.2)* | *6 (54.5)* | *0.916* |  |
| ***SAMA*** | *28 (35.9)* | *23 (34.3)* | *5 (45.5)* | *0.740* |  |
| ***LABA*** | *62 (79.5)* | *53 (85.5)* | *9 (81.8)* | *0.914* |  |
| ***LAMA*** | *51 (65.4)* | *43 (64.2)* | *8 (72.7)* | *0.814* |  |
| ***ICS*** | *59 (75.6)* | *51 (76.1)* | *8 (72.7)* | *0.871* |  |
| ***Theophylline*** | *7 (9.0)* | *7 (10.4)* | *0* | *0.481* |  |
| ***Prednisone^1^*** | *10 (12.8)* | *6 (9.0)* | *4 (36.4)* | *0.040* |  |
| ***Antibiotic^1^*** | *4 (5.1)* | *2 (3.0)* | *2 (18.2)* | *0.100* | |
| ***LTOT*** | *30 (38.5)* | *25 (37.3)* | *5 (45.5)* | *0.820* | |
| **CCI**  **CVD**  **DM/metabolic syndrome**  **Cancer disease**  **CI and Psych D**  **Renal failure and UTD**  **Infect and Immu comp**  **Muscular and skeletal D**  **GI-tract diseases** | 2.0(1.0-3.0)  44(56.4)  22(28.2)  13(16.7)  20(26.6)  13(16.7)  14(17.9)  24(30.8)  25(32.1) | 2.0(1-3)  31(46.2)  19(24.4)  9(11.5)  19(24.4)  12(17.9)  10(12.8)  22(28.2)  18(23.1) | 3.0(2-4)  8(10.3)  3(3.8)  4(5.1)  1(1.3)  1(1.3)  4(5.1)  2(2.6)  7(9.0) | *0.041*  *0.239*  *0.941*  *0.059*  *0.175*  *0.467*  *0.086*  *0.329*  *0.015* | |

Categorical data are presented as n(%), non-parametric data as median (IQR) and parametric data as mean ± (SD). Chi-X^2^, Kruskal-wallis test and T-test were used for statistical analysis respectively.

Abbrevations: Post-BD: post bronchodilator; FEV1: forced expiratory volume in the first second; FVC: Forced expiratory vital capacity; TLC: Totoal lung capacity; RV: Residual volume; TLCO/VA: difusing capacity for carbon monxidedivided by alveolar volume; SABA: short-acting β_2_-agonists; SAMA: short-acting anticholinergic; LABA: long-acting β_2_-agonists; LAMA: long-acting anticholinergic; ICS: Inhaled glucocorticoids; LTOT: Long-term Oxygen therapy; CCI: Charlson Comorbidity Index. CVD: cardiovascular diseases; DM: diabetes mellitus: CI: cognitive impairment; Psych D: psychological disturbances; UTD Urine tract diseases; D: diseases; Infect and Immu comp: (pulmonary) Infectious diseases or immune compromised; GI: gastro intestinal.

^1^ pre-existing maintenance treatment.

**Table 2.** Characteristics at emergency room presentation and non-invasive ventilation (NIV) results of patients hospitalized for ECOPD with acute respiratory failure and requiring non-invasive ventilation (NIV) at first, stratified by in-hospital mortality.

| **Characteristics (n)** | **All**  **(n=78)** | **Hospital survivors**  **(n=67)** | **Hospital**  **deaths**  **(n=11)** | **p-value** |
| --- | --- | --- | --- | --- |
| ***Vital signs***  ***Temperature, ℃ (n=72/61/11)***  ***Temperature, >38 ℃***  ***Systolic BP, mmHg (n=77)***  ***Diastolic BP, mmHg (n=77)***  ***Pulse, beats/min (n=77)***  ***Pulse, >100/min***  ***SpO_2_, % (n=75)***  ***Additional Oxygen (n=73)*** | *37.0±0.93*  *10 (12.8)*  *142.3±34.2*  *77.9±20.1*  *105(95.0-117.5)*  *43 (55.1)*  *90.0 (82-94)*  *44 (60.3)* | *37.1±0.97*  *9 (13.4)*  *143.1±34.4*  *79.4±20.1*  *105.0(93.8-116.3)*  *36 (53.7)*  *90.0(80.8-94.0)*  *28 (43.8)* | *37.0±0.7*  *1 (9.1)*  *137.0±34.1*  *69.5±18.8*  *105.0(95.0-120.0)*  *7 (63.6)*  *91.0(82.0-94.0)*  *5 (45.5)* | *0.898*  *0.513*  *0.585*  *0.132*  *0.021*  *0.784*  *0.010*  *0.280* |
| ***Physical examination***  ***Respiratory rate, /min***  ***(n=42)***  ***Impaired consciousness***  ***(n=76)***  ***Peripheral edema (n=69)***  ***Wheezing (n=76)***  ***Use accessory respiratory***  ***muscle (n=26)***  ***Crackles (n=75)***  ***Elevated CVP (n=30)***  ***BMI, kg/m^2^ (n=74)*** | *30(24-34.3)*  *15 (19.2)*  *28 (35.9)*  *44 (56.4)*  *17 (21.8)*  *32 (41.0)*  *8 (10.3)*  *23.4±(5.2)* | *30.0(23.5-33.0)*  *14 (21.5)*  *26 (38.8)*  *39 (58.2)*  *16 (23.9)*  *29 (43.3)*  *7 (10.4)*  *23.9±5.4* | *31.4(24.5-39.0)*  *1 (9.1)*  *2 (18.2)*  *5 (45.5)*  *1 (9.1)*  *3 (27.3)*  *1 (9.1)*  *20.6±2.2* | *0.683*  *0.337*  *0.337*  *0.292*  *0.483*  *0.433*  *0.386*  *0.002* |
| ***Laboratory results***  ***Hemoglobin, mmol/L***  ***WBC, 10^9^/L***  ***Na, mmol/L***  ***Urea, mmol/L (n=76)***  ***Urea ≥8 mmol/L***  ***Creatinine, mmol/L***  ***Creatinine >115 µmol/L***  ***CRP, mg/L***  ***CRP >50 mg/l***  ***Glucose, mmol/L (n=74)***  ***Troponine ≥0.01 (n=38/32/6)*** | *8.7±1.2*  *14.5±8.5*  *137(134.0-139.3)*  *7.4(5.0-10.3)*  *33 (43.4)*  *85(76.8-90.5)*  *18 (23.1)*  *56.6(24.0-121.0)*  *40 (51.3)*  *9.4±4.7*  *17 (44.7)* | *8.6±1.1*  *13.4±5.8*  *137(134.0-140.0)*  *7.0(4.8-10.0)*  *25 (38.5)*  *84.0(63.0-105.0)*  *13 (19.4)*  *49.0(21.0-103.0)*  *33 (49.3)*  *9.4±4.0*  *12 (37.5)* | *8.4±1.1*  *20.9±16.7*  *136.0(134.0-137.0)*  *10.0(7.7-16.0)*  *8 (72.7)*  *106.0(81.0-137.0)*  *5 (45.5)*  *96.0(31.0-135.0)*  *7 (63.6)*  *9.6±7.6*  *5 (83.3)* | *0.500*  *0.168*  *0.968*  *0.037*  *0.025*  *0.082*  *0.057*  *0.305*  *0.376*  *0.908*  *0.038* |
| ***Chest X-ray (n=77)***  ***Consolidation***  ***Signs of congestion***  ***ECG (n=67)***  ***Atrial fibrillation***  ***Signs of ischemia***  ***p-pulmonale*** | *25 (32.1)*  *12 (15.4)*  *8 (10.3)*  *4 (6.0)*  *13 (16.7)* | *20 (29.9)*  *10 (14.9)*  *7 (12.3)*  *2 (3.5)*  *11 (19.3)* | *5 (50.0)*  *2 (20.0)*  *1 (10.0)*  *2 (20.0)*  *2 (20.0)* | *0.204*  *0.680*  *0.837*  *0.042*  *0.959* |
| **ABG before NIV (n=77)**  **pH**  **<7,25**  **7,25 - 7,35**  **PaCO_2_, kPa**  ***PaCO_2_ >9.1 kPa***  ***HCO_3_^-^ ≥27,0 mmol/L***  **HCO_3_^-^, mmol/L**  **BE**  **<-2,5**  **-2,5 - 2,5**  **>2,5**  ***PaO2, kPa***  ***SaO_2_, %***  ***SaO_2_ <90%***  ***PaO_2_ <7.5 kPa*** | 7.27(7.22-7.31)  28 (36.4)  49 (63.6)  10.0(8.5-11.2)  *50 (64.9)*  *61 (79.2)*  31.8(27.6-36.3)  3.4(-1.0-6.6)  14 (18.2)  21 (27.3)  42 (54.5)  *8.6(7.2-10.7)*  *89.0 (84.0-93.0)*  *39 (54.9)*  *22 (28.6)* | 7.28(7.22-7.31)  21 (31.8)  45 (68.2)  10.0(8.5-11.0)  *42 (63.6)*  *56 (84.8)*  31.9(28.0-36.2)  3.7(-0.3-6.6)  9 (13.6)  21 (31.8)  36 (54.5)  *8.6(7.4-10.5)*  *89.0(84.0-92.0*  *34 (55.7)*  *18 (27.3)* | 7.24(7.1-7.26)  7 (63.6)  4 (36.4)  10.8(8.3-12.1)  *8 (72.7)*  *5 (54.5)*  26.7(25.4-36.8)  2.9(-5.4-6.6)  5 (45.5)  0  6 (54.5)  *9.6(6.3-12.3)*  *90.0(72.5-96.0)*  *5 (14.1)*  *4 (36.4)* | *0.015*  *0.042*  *0.322*  0.559  *0.003*  *0.799*  *0.256*  *0.013*  0.799  0.980  0.735  0.537 |
| ***Policy restrictions***  ***Assessed before admission***  ***Changed at admission***  ***Present before NIV*** | *14(17.9)*  *36(46.2)*  *46(59.0)* | *12 (17.9)*  *26 (38.8)*  *36 (53.7)* | *2 (18.2)*  *10 (90.9)*  *10 (90.9)* | *0.983*  *0.001*  *0.020* |
| **Clinical data** |  |  |  |  |
| **LOS, days**  **NIV, days**  **NIV ≤8 days**  **IPAP, cm H_2_O**  **EPAP, cm H_2_O** | 16.5(11.0-28.25)  5.0(2.8-7.0)  63 (85.9)  20.8±4.3  6.4±1.5 | 17.0(11.0-27.0)  5.0(2.0-7.0)  56 (83.6)  20.5±4.3  6.2±1.5 | 11.0(3.0-31.0)  7.0(3.0-11.0)  7 (63.6)  22.9±3.8  7.1±1.4 | *0.438*  *0.337*  *0.120*  *0.081*  *0.087* |
| **NIV response after 1h**  **Delta pCO_2_ before NIV-1h,kPa**  **pH ≥7.35 at 1h**  **RR at 1h, /min**  **RR >20/min at 1h**  **NIV response after 24h**  **Delta PaCO_2_ before NIV-24h, kPa**  **pH ≥7.35 24h (n=63)**  **RR 24h, /min (n=56)**  **RR >20/min at 24h**  **NIV response at stop NIV**  **Delta PaCO_2_ before NIV-**  **stop, kPa**  **Delta bicarbonate before NIV-stop, mmol/l**  **BE at stop NIV**  **Delta BE before NIV-stop**  **(n=74)**  **Successful NIV**  **Tolerance NIV** | 1.4±1.2  22 (28.6)  22.8±5.9  39 (61.9)  2.1±2.2  35 (55.6)  21.2±4.5  25 (44.6)  2.3±2.9  -5.2±-0.9  7.6±13.3  5.0±13.0  43 (55.1)  55 (73.3) | 1.4±1.3  21 (31.8)  22.2±5.9  31 (57.4)  2.1±2.3  32 (60.4)  21.4±4.6  22 (44.9)  2.4±2.2  -6.0±42.0  8.6±13.8  5.8±13.5  40 (59.7)  54 (98.2) | 1.6±1.1  1 (9.1)  26.7±4.4  8 (88.9)  2.2±1.9  3 (30.0)  19.9±3.6  3 (42.9)  1.8±3.4  0.4±7.2  1.1±5.6  0.1±8.7  3 (27.3)  1 (1.8) | *0.548*  *0.122*  *0.034*  *0.072*  *0.888*  *0.076*  *0.401*  *0.919*  *0.519*  *0.631*  *0.099*  *0.201*  *0.045*  *0.000* |

Categorical data are presented as n(%), non-parametric data as median (IQR) and parametric data as mean ± (SD). Chi-X^2^, Kruskal-wallis test and T-test were used for statistical analysis respectively.

Abbrevations: BP: blood pressure; CVP: central venous pressure; BMI: body mass index; NIV: non-invasive ventilation; WBC: white blood cell count, Na: Sodium; ECG: electrocardiogram; ABG: arterial blood gas; LOS: Length of hospital stay; IPAP: inspiratory positive air pressure; EPAP: Expiratory positive air pressure; RR: respiratory rate; BE: base excess; NIV: non-invasive ventilation.

**Table 3.** Characteristics of patients hospitalized for ECOPD with acute respiratory failure and requiring non-invasive ventilation at first, stratified by 2-year outcome.

|  | **All**  **(n=78)** | **Long-term survivors**  **(n=34)** | **Long-term deaths**  **(n=44)** | **p-value** |
| --- | --- | --- | --- | --- |
| **Male gender** | 38 (48.7) | 11 (28.9) | 27 (71.1) | *0.011* |
| **Age at admission, y** | 71.0±10.7 | 66.9±11.6 | 74.1±8.9 | *0.003* |
| ***Smoking status*** |  |  |  |  |
| ***Non smoker*** | *1 (1.3)* | *0* | *1 (1.3)* | *0.176* |
| ***Former smoker*** | *32 (41.0)* | *13 (38.2)* | *19 (43.2)* |  |
| ***Current smoker*** | *41 (52.6)* | *21 (61.8)* | *20 (45.5)* |  |
| ***Unknown*** | *4 (5.1)* | *0* | *4 (9.1)* |  |
| **Pack years (n=32;18;14)** | 43.5±12.3 | 39.2±12.4 | 48.9±10.2 | *0.024* |
| ***GOLD stage*** |  |  |  |  |
| ***Gold II*** | *16 (20.5)* | *8 (23.5)* | *8 (18.2)* |  |
| ***Gold III*** | *21 (26.9)* | *9 (26.5)* | *12 (27.3)* |  |
| ***Gold IV*** | *40 (51.3)* | *16 (47.1)* | *24 (54.5)* | *0.620* |
| ***Post-BD* FEV_1_, %pred** | 39.0(28.6-52.9) | 40.2(29.9-54.3) | 36.9(27.2-49.8) | *0.351* |
| ***Post-BD* *FEV_1_, L*** | *0.9±0.5* | *0.9±0.5* | *1.0±0.46* | *0.908* |
| ***FEV_1_/FVC*** | *38.3±11.2* | *39.4±12.1* | *37.4±10.4* | *0.417* |
| ***RV/TLC, % (n=53;23;30)*** | *61.9±10.0* | *59.4±11.8* | *63.9±8.1* | *0.105* |
| ***TLCO/VA, %pred (n=51;23;28)*** | *51.1±21.8* | *54.6±25.0* | *48.2±18.7* | *0.305* |
| ***Hospitalization for ECOPD previous first year*** | *26 (33.3)* | *8 (23.5)* | *18 (56.4)* | *0.106* |
| ***0-1***  **Frequent exacerbators (>1)** | *67 (85.9)*  11 (14.1) | *30 (44.8)*  4 (11.8) | *37 (55.2)*  7 (15.9) | *0.602* |
| ***Living status n=75***  ***At Home***  ***Alone***  ***Alone with care***  ***Cohabitation***  ***Cohabitation with care*** | *65 (83.3)*  *10 (12.8)*  *20 (25.6)*  *10 (12.8)*  *25 (32.1)* | *30 (88.2)*  *5 (14.7)*  *11 (32.4)*  *7 (20.6)*  *7 (20.6)* | *36 (85.7)*  *5 (11.6)*  *9 (20.9)*  *3 (7.0)*  *18 (41.9)* | *0.746*  *0.285* |
| ***Resident (not at home)*** | *10 (14.1)* | *4 (11.8)* | *6 (14.3)* | *0.746* |
| ***No additional care*** | *20 (25.6)* | *12 (35.3)* | *8 (19.5)* | *0.124* |
| ***Treatment for COPD yes*** | *73 (93.6)* | *30 (88.2)* | *43 (97.7)* | *0.214* |
| ***SABA*** | *43 (55.1)* | *20 (58.8)* | *23 (52.3)* | *0.399* |
| ***SAMA*** | *28 (35.9)* | *14 (41.2)* | *14 (31.8)* | *0.329* |
| ***LABA*** | *62 (79.5)* | *23 (67.6)* | *39 (88.6)* | *0.061* |
| ***LAMA*** | *51 (65.4)* | *17 (50.0)* | *34 (77.3)* | *0.032* |
| ***ICS*** | *59 (75.6)* | *25 (73.5)* | *34 (77.3)* | *0.513* |
| ***Theophylline*** | *7 (9.0)* | *4 (11.8)* | *3 (6.8)* | *0.377* |
| ***Prednisone^1^*** | *10 (12.8)* | *1 (2.9)* | *9 (20.5)* | *0.04* |
| ***Antibiotics^1^*** | *4 (5.1)* | *0* | *4 (9.1)* | *0.107* |
| ***LTOT*** | *30 (38.5)* | *10 (29.4)* | *20 (45.5)* | *0.210* |
| **CCI**  **CVD**  **DM/metabolic syndrome**  **Cancer disease**  **CI and Psych D**  **Renal failure and UTD**  **Infect or immu comp**  **Muscular and skeletal D**  **GI-tract diseases** | 2.0 (1.0-3.0)  44(56.4)  22(28.2)  13(16.7)  20(25.6)  13(16.7)  14(17.9)  24(30.8)  25(32.1) | 2 (1-3)  17(21.8)  10(29.4)  4(5.1)  10(12.8)  7(9.0)  4(5.1)  9(11.5)  5(6.4) | 2 (1-3)  27(34.6)  12(15.4)  9(11.5)  10(12.8)  6(7.7)  10(12.8)  15(19.2)  20(26.6) | *0.478*  *0.316*  *0.835*  *0.307*  *0.503*  *0.414*  *0.211*  *0.470*  *0.004* |

Categorical data are presented as n(%), non-parametric data as median (IQR) and parametric data as mean ± (SD). Chi-X^2^, Kruskal-wallis test and T-test were used for statistical analysis respectively.

Abbrevations: : Post-BD: post bronchodilator; FEV1: forced expiratory volume in the first second; FVC: Forced expiratory vital capacity; TLC: Totoal lung capacity; RV: Residual volume; TLCO/VA: difusing capacity for carbon monxidedivided by alveolar volume; SABA: short-acting β_2_-agonists; SAMA: short-acting anticholinergic; LABA: long-acting β_2_-agonists; LAMA: long-acting anticholinergic; ICS: Inhaled glucocorticoids; LTOT: Long-term Oxygen therapy; CCI: Charlson Comorbidity Index. CVD: cardiovascular diseases; DM: diabetes mellitus: CI: cognitive impairment; Psych D: psychological disturbances; UTD Urine tract diseases; D: diseases; Infect and Immu comp: (pulmonary) Infectious diseases or immune compromised; GI gastro intestinal.

^1^ pre-existing maintenance treatment.

**Table 4.** Characteristics at emergency room presentation and non-invasive ventilation (NIV) results of patients hospitalized for ECOPD with acute respiratory failure and requiring non-invasive ventilation at first, stratified by 2-year outcome.

| **Characteristics (n)** | **All**  **(n=78)** | **Long-term survivors**  **(n=34)** | **Long-term deaths**  **(n=44)** | **p-value** |
| --- | --- | --- | --- | --- |
| ***Vital signs***  ***Temperature,℃ (n=72/31/41)***  ***Temperature >38℃***  ***Systolic BP, mmHg (n=77)***  ***Diastolic BP, mmHg (n=77)***  ***Pulse, beats/min (n=77)***  ***Pulse >100/min***  ***SpO_2_, %(n=75)***  ***Additional oxygen (n=73)*** | *37.1(36.5-37.5)*  *10 (12.8)*  *142.3±34.2*  *77.9±20.1*  *105(95.0-117.5)*  *43 (55.1)*  *90.0(82-94)*  *44 (60.3)* | *37.1±1.0*  *6 (17.6)*  *128.1±29.0*  *73.4±14.9*  *95.0(84.8-115.0)*  *16 (47.1)*  *91.0(83.5-94.8)*  *18 (58.1)* | *36.9±0.8*  *4 (9.1)*  *153.4±33.5*  *81.5±23.0*  *110.0(95.0-120.0)*  *27 (61.4)*  *90.0(80.0-94.0)*  *26 (61.9)* | *0.194*  *0.481*  *0.001*  *0.066*  *0.116*  *0.260*  *0.443*  *0.497* |
| ***Physical examination***  ***Respiratory rate/min (n=42)***  ***Impaired consciousness (n=76)***  ***Peripheral edema (n=69)***  ***Wheezing (n=76)***  ***Use accessory respiratory muscle (n=26)***  ***Crackles (n=75)***  ***Elevated CVP (n=30)***  ***Body mass index, kg/m^2^*** | *30.0(24-34.3)*  *15 (19.2)*  *28 (35.9)*  *44 (56.4)*  *17 (21.8)*  *32 (41.0)*  *8 (10.3)*  *23.4*±5.2 | *25.0(22.5-30.0)*  *5 (14.7)*  *12 (35.3)*  *21 (61.8)*  *10 (29.4)*  *14 (41.2)*  *4 (11.8)*  24.8±5.6 | *30.0(27.5-35.0)*  *10 (22.7)*  *16 (36.4)*  *23 (52.3)*  *7 (15.9)*  *18 (40.9)*  *4 (9.1)*  22.3±4.7 | *0.26*  *0.383*  *0.345*  *0.663*  *0.204*  *0.935*  *0.888*  0.41 |
| ***Chest X-ray (n=77)***  ***Consolidation***  ***Signs of congestion***  ***ECG (n=67)***  ***Atrial fibrillation***  ***Signs of ischemia***  ***p-Pulmonale*** | *25 (32.1)*  *12 (15.4)*  *8 (10.3)*  *4 (5.1)*  *13 (16.7)* | *8 (23.5)*  *4 (11.8)*  *1 (3.4)*  *1 (3.4)*  *6 (20.7)* | *17 (39.5)*  *8 (18.6)*  *7 (18.4)*  *4 (7.9)*  *7 (18.4)* | *0.136*  *0.411*  *0.061*  *0.447*  *0.816* |
| **ABG before start NIV (n=77)**  **pH**  **<7,25**  **7,25 - 7,35**  **PaCO_2_, kPa**  ***PaCO_2_ >9.1 kPa***  ***HCO_3_^-^ ≥27,0 mmol/L***  **HCO_3_^-^, mmol/L**  **Base excess**  **< -2,5**  **-2,5 - 2,5**  **>2,5**  ***PaO_2_, kPa***  ***SaO_2_, %***  ***SaO_2_ <90%***  ***PaO_2_ <7.5 kPa*** | 7.27(7.22-7.31)  28 (36.4)  49 (63.6)  10.0(8.5-11.2)  *50 (64.9)*  *61 (79.2)*  31.8(27.6-36.3)  3.4(-1.0-6.6)  14 (18.2)  21 (27.3)  42 (54.5)  *8.6(7.2-10.7)*  *89.0 (84.0-93.0)*  *39 (54.9)*  *22 (28.6)* | 7.28(7.23-7.31)  8 (23.5)  26 (76.5)  9.7(8.5-10.5)  *20 (58.8)*  *30 (88.2)*  31.4(27.8-73.0)  3.9(-0.3-7.0)  3 (8.8)  12(35.3)  19 (55.9)  *8.5(7.2-10.5)*  *89.0(85.0-93.0)*  *16 (51.6)*  *11 (32.4)* | 7.25(7.18-7.29)  20 (46.5)  23 (53.5)  10.3(8.6-11.5)  *30 (69.8)*  *31 (72.1)*  31.8(26.7-36.0)  3.1(-2.6-6.5)  11(25.6)  9 (20.9)  23 (53.5)  *8.8 (7.0-10.7)*  *88.0 (80.0-93.0)*  *23 (57.5)*  *11 (25.6)* | *0.024*  *0.037*  *0.270*  *0.318*  0.083  0.538  *0.340*  *0.111*  0.955  0.260  0.621  0.514 |
| ***Laboratory results***  ***Hemoglobin mmol/L***  ***WBC 10^9^/L (362/331/31)***  ***Na mmol/L***  ***Urea mmol/L (n=76)***  ***Creatinine mmol/L***  ***Creatinine >115 µmol/L***  ***CRP mg/L***  ***CRP >50 mg/l***  ***Glucose mmol/L (n=74)*** | *8.7±1.2*  *14.5±8.5*  *137(134-139.3)*  *7.4(5.0-10.3)*  *85(76.8-90.5)*  *18 (23.1)*  *56.6(24.0-121.0)*  *40(51.3)*  *9.4±4.7* | *8.9±0.9*  *13.2±5.8*  *137(134.0-139.0)*  *7.3(4.3-9.7)*  *85(66.5-107.0)*  *7(20.6)*  *75.0(11.5-153.0)*  *19(55.9)*  *7.8(6.4-10.3)* | *8.6±1.3*  *15.4±10.1*  *137.0(134.0-140.0)*  *7.7(6.2-10.6)*  *84.5(63.5-114.0)*  *11(25.0)*  *46.0(25.5-94.0)*  *21(47.7)*  *7.6(6.6-11.3)* | *0.244*  *0.264*  *0.907*  *0.314*  *0.892*  *0.647*  *0.268*  *0.475*  *0.756* |
| ***Limited policy restrictions***  ***Assessed before admission***  ***changed at admission***  ***present before NIV*** | *14 (17.9)*  *36 (46.2)*  *46 (59.0)* | *5 (14.7)*  *8 (23.5)*  *13 (38.2)* | *9 (20.5)*  *28 (63.6)*  *33 (75.0)* | *0.512*  *0.000*  *0.001* |
| **Clinical data** |  |  |  |  |
| **LOS, days**  **NIV, days**  **NIV >8 days**  **IPAP, cm H_2_O**  **EPAP, cm H_2_O** | 16.5(11.0-28.25)  5.0(2.8-7.0)  15 (19.2)  20.8±4.3  6.4±1.5 | 13.5(10.0-18.3)  4.0(3.0-6.0)  3 (8.8)  21.2±3.9  6.3±1.3 | 17.0(8.3-28.0)  5.0(2.0-10.8)  12 (27.3)  20.6±4.5  6.4±1.7 | *0.528*  *0.248*  *0.040*  *0.535*  *0.744* |
| **NIV response after 1h**  **Delta PaCO_2_ before NIV-1h, kPa**  **pH ≥7.35 at1h**  **RR at 1h, /min**  **RR >20/min at 1h**  **NIV response after 24h**  **Delta pCO_2_ before NIV-24h,**  **kPa**  **pH ≥7.35 24h (n=63)**  **RR 24h, /min (n=56)**  **RR >20/min at 24h**  **NIV response at stop NIV**  **Delta before NIV-stop PaCO_2_, kPa**  **Delta bicarbonate before NIV-stop, mmol/l**  **BE at stop NIV**  **Delta BE before NIV-stop (n=74)**  **Successful NIV**  **Tolerance/ adherence NIV** | 1.4±1.2  22 (28.6)  22.8±5.9  39 (61.9)  2.1±2.2  35 (55.6)  21.2±4.5  25 (44.6)  2.3±2.9  -5.2±-0.9  7.6±13.3  5.0±13.0  43(55.1)  55(73.3) | 1.1±1.1  12 (35.3)  21.0±6.1  14 (48.3)  2.0±1.9  16 (59.3)  20.4±3.7  9 (34.6)  2.6±17.5  -10.8±57.5  10.7±18.6  7.3±18.0  20(58.8)  28(82.4) | 1.6±1.3  10 (23.3)  24.3±5.4  25 (73.5)  2.3±2.4  19 (52.8)  21.9±5.1  16 (53.3)  -2.8±2.9  -0.3±5.4  5.0±5.2  6.2±5.9  23(52.3)  27(65.9) | *0.094*  *0.246*  *0.027*  *0.040*  *0.669*  *0.608*  *0.204*  *0.160*  *0.058*  *0.252*  *0.067*  *0.180*  *0.564*  *0.108* |

Categorical data are presented as n(%), non-parametric data as median (IQR) and parametric data as mean ± (SD). Chi-X^2^, Kruskal-wallis test and T-test were used for statistical analysis respectively.

Abbrevations: ABG: arterial blood gas; LOS: Length of hospital stay; IPAP inspiratory positive air pressure; EPAP: Expiratory positive air pressure; RR: respiratory rate; BE: base excess; NIV: non-invasive ventilation.

**Table 5.** Readmission data in study population during two years follow-up.

Data presented as n(%) or abnormal distribution median (IQR)

| **Readmission data** | **n=67** | |
| --- | --- | --- |
| **Total number readmissions <2 years**  **1 readmission <2 years**  **2 readmissions <2 years**  **3 or more readmissions <2 years**  **H-ECOPD free-interval days**  **Combined dead or H-ECOPD <1year**  **Combined dead or H-ECOPD <2 years**  **Readmission NIV needed <2 years**  **1**  **2 or more**  **Readmission IV needed <2 years (once)** | | 83 (times)  10 (14.9)  10 (14.9)  12 (17.9)  124.5 (32.8-239.8)  39 (58.2)  44 (65.7)  38 (times)  10 (14.9)  10 (14.9)  6 (9.0) |

Abbrevations: H-ECOPD: Hospitalised exacerbation of COPD; NIV: non-invasive mechanical ventilation: IV: invasive mechanical ventilation
